# The Influence of Different EEG References on Scalp EEG Functional Network Analysis During Hand Movement Tasks

**DOI:** 10.3389/fnhum.2020.00367

**Published:** 2020-09-02

**Authors:** Lipeng Zhang, Peng Wang, Rui Zhang, Mingming Chen, Li Shi, Jinfeng Gao, Yuxia Hu

**Affiliations:** ^1^School of Electrical Engineering, Zhengzhou University, Zhengzhou, China; ^2^Henan Key Laboratory of Brain Science and Brain-Computer Interface Technology, Zhengzhou, China; ^3^Department of Automation, Tsinghua University, Beijing, China; ^4^Beijing National Research Center for Information Science and Technology, Beijing, China

**Keywords:** EEG, common average reference, reference electrode standardization technique, scalp EEG functional network, hand movement tasks

## Abstract

Although scalp EEG functional networks have been applied to the study of motor tasks using electroencephalography (EEG), the selection of a suitable reference electrode has not been sufficiently researched. To investigate the effects of the original reference (REF-CZ), the common average reference (CAR), and the reference electrode standardization technique (REST) on scalp EEG functional network analysis during hand movement tasks, EEGs of 17 right-handed subjects performing self-paced hand movements were collected, and scalp functional networks [coherence (COH), phase-locking value (PLV), phase lag index (PLI)] with different references were constructed. Compared with the REF-CZ reference, the networks with CAR and REST references exhibited more significant increases in connectivity during the left-/right-hand movement preparation (MP) and movement execution (ME) stages. The node degree of the channel near the reference electrode was significantly reduced by the REF-CZ reference. CAR and REST both decreased this reference effect, REST more so than CAR. We confirmed that the choice of reference would affect the analysis of the functional network during hand movement tasks, and the REST reference can greatly reduce the effects of the online recording reference on the analysis of EEG connectivity.

## Introduction

Maintaining body movement ability is one of the most important functions of the brain, and many researchers have devoted themselves to studying the mechanisms behind motor processes. Electroencephalography (EEG) is a non-invasive approach with a high temporal resolution that is widely used to study neural activity during motor tasks.

After Kornhuber and Deecke first discovered the “Bereitschaftspotential” (BP; Kornhuber and Deecke, 1965; Shibasaki and Hallett, 2006), several studies on movement-related cortical potentials (MRCP) have been reported (Tarkka and Hallett, 1991; Rong and Deecke, 1999; Berchicci et al., 2016). For simple movements, the BP initiates for 1–2 s and abruptly begins to increase at 0.5 s before the movement onset over the frontal-central areas of the scalp (Rong and Deecke). The power variation of the relevant frequency bands during movement has been extensively studied (Pfurtscheller, 1990; Pfurtscheller et al., 2003; Bai et al., 2005). Pfurtscheller (1990) studied the event-related desynchronization/synchronization (ERD/ERS) of various frequency bands associated with voluntary movement. Their research demonstrated ERD over contralateral and ipsilateral motor cortices during the movement preparation (MP) and movement execution (ME). Recent developments in functional connectivity research provided a new method for neuroimaging (Biswal et al., 1995). Complex brain networks provide some quantitative indicators for the topology of brain networks and have become important tools for describing anatomical and functional brain connectivity (Bullmore and Sporns, 2009; Rubinov and Sporns, 2010). Although some questions remain to be solved, this new method will help us understand the mechanisms behind motor processes (Sporns et al., 2004). Recently, brain network methods have been utilized to analyze changes in network connectivity between brain regions (Popovych et al., 2016; Fleck et al., 2018). Brain connectivity analysis has also been used as a feature extraction method (Li et al., 2018b).

The choice of the reference electrode is an inevitable issue in EEG research. But there is still debate about which is the most suitable reference (Yao et al., 2019). All signals of EEG in each electrode are obtained as the difference between the electric potentials in its location and in the location of the reference electrode. However, it is impossible to find a neutral reference on the scalp or body. To solve this problem, many studies have aimed to find a relatively non-active point on the body surface (Offner, 1950; Yao, 2001; Hagemann et al., 2001). Common online recording references include the FZ, CZ, PZ, OZ left/right earlobe, and nose (Andrew and Pfurtscheller, 1996; Bruder et al., 1997; Başar et al., 1998; Essl and Rappelsberger, 1998; Hu et al., 2018a). Offline re-references mainly include the linked ears/mastoids (Garneski and Steelman, 1958), the common average reference (CAR; Offner, 1950), the reference electrode standardization technique (REST; Yao, 2001) and the unified referencing framework (rREST; Hu et al., 2018b). The family of EEG unipolar references including REF-Cz, CAR, and REST, etc. has the “no memory” property (Hu et al., 2019). Because of this, we can always re-reference the dataset. Although the brain network method has been applied to the study of motor tasks, the problem of selecting an appropriate reference electrode in EEG research has not been solved (Popovych et al., 2016; Storti et al., 2016; Li et al., 2018b). Similarly, the problem of the selection of the reference electrode also exists in the motor imagination (MI) that have similar pattern of brain networks with the ME (Zhang et al., 2015; Li et al., 2018a, 2019). Recently, some studies investigated how different reference choices influence scalp EEG functional connectivity using simulated EEG data (Chella et al., 2016; Huang et al., 2017). These studies all pointed out that different references significantly alter the topography of EEG connectivity patterns. Although the influence of the reference electrode on MRCP has been studied (Hu et al., 2017), its effect on the brain network of motor tasks has not been fully discussed.

Hand movement tasks rely on multiple brain regions working together and consist of motor preparation and motor execution processes. The present study aimed to investigate the influence of different references (REF-CZ, CAR, and REST) on brain network analysis for motor tasks. First, we divided motor tasks into two phases based on ERD in the beta band: motor preparation and motor execution. Functional brain networks were then constructed with different re-referencing schemes in the beta band. Finally, we compared connectivity variations and the graph-theoretic measures of functional brain networks during motor tasks using different re-referencing schemes.

## Materials and Methods

### Subjects

A total of seventeen right-handed healthy subjects (one female; mean age 26 years, range: 23–29 years) were recruited from Zhengzhou University. They had a normal or corrected-to-normal vision, and none of them had a history of motor or neurological disease. Every subject was informed of the experimental procedure and signed a letter of consent before the experiment. The study was approved by the local ethics committee for the Protection of Human Subjects at Zhengzhou University.

### Procedure and Task

Each subject was comfortably seated in front of a computer screen and remained idle, with their hands, forearms, and elbows resting on the armrest of the chair. During EEG recording, each subject was asked to try to avoid eye movement, swallowing, and unnecessary limb movements.

**Figure 1:**
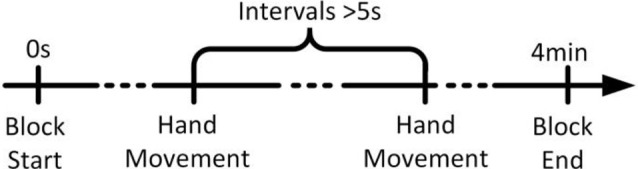
The timeline of a run. Each trial began in the idle stage, in which the subject rested his or her hands, forearms, and elbows on the armrest of a chair and relaxed his or her hands. The subject performed each hand movement task after taking more than 5 s of idle time. After the completion of a task, the subject returned to the idle stage and prepared for the next task.

As shown in Figure 1, subjects performed self-paced hand movement tasks that required intervals of more than 5 s between each task. Subjects were free to choose their left or right hands for each movement, but the number of left-/right-hand movements was approximately the same. They were instructed not to count the number of seconds in an interval, and we emphasized the importance of movement intention immediately before performing the movement. The recording was done in ten 4 min long blocks with intermittent 2 min breaks.

### EEG Recording and Signal Preprocessing

EEG data were recorded by a Neuroscan NuAmps digital amplifier system with 64 electrodes arranged in the standard 10–10 EEG configuration. All the brain regions were covered by these electrodes. Two extended bipolar channels (BP3 and BP4) were used to acquire the left and right arms’ EMG (electromyography) signals. The EEG signals were acquired at a sampling rate of 250 Hz with the REF-CZ as an online recording reference, and the impedance of all electrodes was less than 5 kΩ. The REF-CZ was located between the CZ and CPZ electrodes.

Data preprocessing consisted of two parts: EMG data and EEG data preprocessing. EMG data were used to obtain the onset time of each hand movement for each trial. The EMG data were band filtered by a basic finite impulse response filter with respective cutoff frequencies of 6 and 50 Hz. We then calculated the energy of the filtered data and set the proper threshold to detect the onset time of movement. Next, we checked the records visually to remove instances of false recognition. Finally, we recorded the onset times in a TXT file (Hu et al., 2017).

The EEGLAB toolbox was used to preprocess and analyze the EEG data (Delorme and Makeig, 2004). First, we imported the time labels of movement onset into the data. Next, the raw EEG data were visually inspected for eye-blinks and muscle artifacts. Then, testing with CAR was conducted using the *reref* function from the EEGLAB toolbox, and testing with REST was conducted using the *rest_refer* function^1^.

To extract the frequency band of interest, the data were band-pass filtered in 13–30 Hz. Then, taking the EMG onset as 0 ms, all EEG data of the three references were segmented into 5,000 ms epochs ranging from 3,000 ms before to 2,000 ms after the onset of movement [i.e., (−3,000, 2,000) ms].

**Figure 2:**
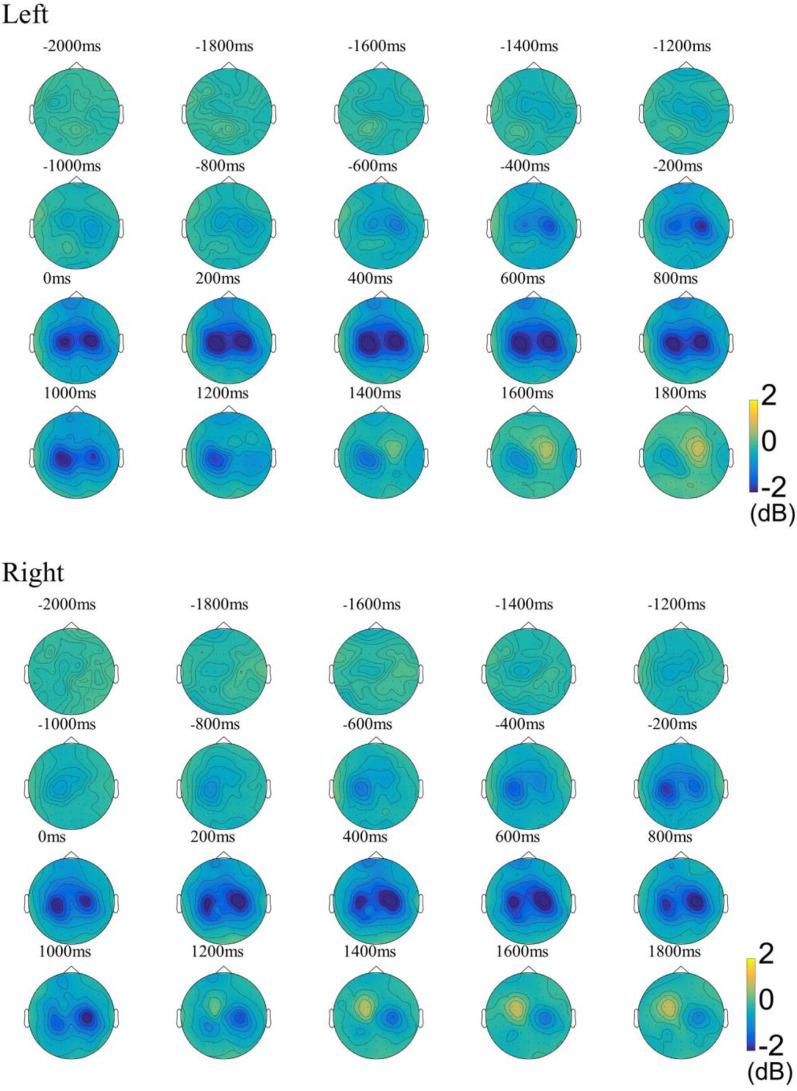
The ground-average event-related desynchronization (ERD) patterns in the beta band (13–30 Hz) during the left-/right-hand movement. The baseline was −3 to −2 s. Blue indicates ERD, yellow indicates event-related synchronization (ERS). Left, Left-hand movement; Right, Right-hand movement.

For examining the ERD patterns in the beta band, we used the FieldTrip toolbox for time-frequency analysis (Oostenveld et al., 2011). Multitaper method (MTM) based Hanning tapers were used to calculate the time-frequency representations (TFRs) of power (Thomson, 1982). Time-frequency representations (TFRs) were calculated using the data with the REST reference due to the theoretical advantages of the REST reference (Yao et al., 2005). As shown in Figure 2, our results showed that ERD was observed in the central areas of the contralateral hemisphere as early as about 1,000 ms before movement onset and continued to enhance. ERD was also observed soon after over the central area on the ipsilateral hemisphere, and its distribution became bilaterally symmetrical from 0 ms concerning movement onset. At about 1,000 ms, weak ERD was observed only on the ipsilateral hemisphere, and ERS begins to appear in the contralateral hemisphere. The results of the ERD distribution in the beta band are consistent with previous research (Pfurtscheller and Aranibar, 1977; Bai et al., 2005). To compute functional network changes, the preprocessed signals were divided into three stages according to the ERD change pattern: idle stage [(−3,000, −2,000) ms], MP stage [(−1,000, 0) ms], and ME stage [(0, 1,000) ms].

### Brain Network Construction

This study used the following most commonly used connectivity measurement methods.

(1) Coherence: The coherence, also referred to as the magnitude squared coherence or coherence spectrum, between two signals is their cross-spectral density function (Pereda et al., 2005). Coherence is a widely-used measure for characterizing linear dependence between a pair of stochastic processes, as well as a quantitative measure of their phase consistency, and may be viewed as the equivalent measure of cross-correlation in the frequency domain. It is defined as

(1) $$COH_{xy}(f)=\frac{{\left|S_{xy}(f)\right|}^{2}}{S_{xx}(f)S_{yy}(f)}$$

where *S_xy_(f)* is the cross-spectrum of the signals *x(t)* and *y(t)*, and *S_xx_(f)* and *S_yy_(f)* are their respective self-spectra. The COH ranges between 0 and 1: 0 ≤ COH ≤ 1.

(2) Phase-Locking Value: The phase-locking value (PLV) makes use only of the relative phase difference (Lachaux et al., 1999). It is defined as

(2) $$PLV=\frac{1}{K}\left|\sum_{K=1}^{K}\exp(j\theta (t_{k}))\right|$$

where *t* is the time, and θ(*t*_k_) is the phase difference Ø_1_(*t*_k_) – Ø_2_(*t*_k_) PLV measures the variability of this phase difference at *t*. If the phase difference varies little across the trials, PLV is close to 1; it is close to zero otherwise.

(3) Phase Lag Index: The phase lag index (PLI) was introduced by Stam et al. (2007), aiming to deal with the problem of volume conduction and active reference electrodes in the assessment of functional connectivity. The central idea is to discard phase differences that center around 0 mod π. It is defined as

(3) $$PLI=\left|\langle sign\left[\Delta \emptyset (t_{k})\right]\rangle \right|$$

The PLI ranges between 0 and 1: 0 ≤ PLI ≤ 1. A PLI of 0 indicates either no coupling or coupling with a phase difference centered around 0 mod π. A PLI of 1 indicates perfect phase locking at a value of ΔØ different from 0 mod π. The stronger this non-zero phase locking, the larger the PLI.

We adopted the above-mentioned three methods to construct scalp functional networks for the idle stage, MP stage, and ME stage of left-/right-hand movement tasks. For every method, we calculated the connectivity value between per electrode pairs and stored all the connectivity values in the adjacency matrix in which the rows and columns are arranged in order of channel number. Then, the adjacency matrix G of each subject has been obtained by averaging the adjacency matrices of all trials for left-/right-hand movement networks from each reference and stage. Finally, 18 adjacency matrices of functional networks of the left-/right-hand movement were calculated from the data of three stages with three references for each subject.

### Network Measures

Graph theory provides a quantitative analysis method for us to analyze complex brain networks. Commonly used network parameters can be roughly divided into local parameters (node degree, clustering coefficient, local efficiency, and so on) and global parameters (characteristic path length, global efficiency, and so on). Node degree is the most fundamental network measure, and most other measures are ultimately linked to the node degree (Bullmore and Sporns, 2009). The characteristic path length is a commonly used global parameter, and most other global parameters are related to it. Therefore, this article selected the two parameters of node degree and characteristic path length.

(1) Degree: the degree of a node is the number of connections involving it (Sporns et al., 2004). It can be defined as follows:

(4) $$D_{i}=\sum_{j\neq i}A_{ij}$$

where *D_i_* is the degree of node *i*, and *A_ij_* represents the connectivity between node *i* and node *j*.

(2) Characteristic path length: path length is the minimum number of edges that must be traversed to go from one node to another. The average length of the shortest paths between per pairs of nodes is the characteristic path length. Thus, it is also called the shortest path length. It can be defined as follows:

(5) $$L=\frac{1}{N(N-1)}\sum_{i,j\in N,i\neq j}d_{ij}$$

Where *d_ij_* is the shortest path (geodesic) between *i* and *j*. Note that *d_ij_* = ∞ for all disconnected pairs *i*, *j*.

### Statistical Analysis

To find out which edges had significantly increased connectivity during the MP and ME stages compared to the idle stage, the three networks of the left-/right-hand movement were constructed with different references. To get the average adjacency matrix for the three stages, we averaged all subjects’ adjacency matrices at each stage. Then the averaged adjacency matrices of the idle stage were subtracted from the averaged adjacency matrices during MP and ME stage. All elements in these difference matrices greater than 0.02 are considered to have significantly increased. To avoid the consideration of spurious interactions, a one-tailed paired *t*-test was used to test the differences in connectivity of all the subjects between two stages. The sample size of the *t*-test was 17 since we had 17 subjects. To reduce the false-positive rate, statistical tests were corrected with the BHFDR method (Benjamini and Hochberg, 1995). If the BHFDR-corrected significance level of an edge was less than 0.05, we considered the corresponding connectivity to have increased significantly and retained the edge, otherwise discard this edge. To compare the network parameters of functional networks with different references, the two-sample paired *t*-test was used to test the differences in degree and characteristic path length between three references (REF-CZ vs. CAR, REF-CZ vs. REST, and CAR vs. REST). The sample size of the *t*-test was 17.

## Results

### Effect of Reference Choice on COH, PLV, and PLI Networks Analysis in Hand Movement Task

The number of edges with significantly increased connectivity during the MP stage and ME stage is shown in Table 1 (*P* < 0.05, BHFDR). A significant increase in connectivity can be observed in COH and PLV networks. The networks with the REF-CZ reference has far fewer edges than the networks with CAR and REST reference during the ME stage. The networks with REST reference has slightly more edges than the networks with CAR reference in both the stages of MP and ME. There was almost no significant increase in connectivity in the PLI networks, which may reflect that non-zero lag phase synchronization between almost all electrode pairs does not increase significantly during MP and ME stages in the beta band.

**Table 1:** The number of edges with significantly increased connectivity during the movement preparation (MP) stage and movement execution (ME) stage.

Connectivity	Movement stage	The number of edges with significantly increased connectivity (*P* < 0.05, BHFDR)		
		REF-CZ	CAR	REST
COH	MP (L)	21	6	17
	ME (L)	5	60	86
	MP (R)	11	16	25
	ME (R)	7	19	46
PLV	MP (L)	28	24	30
	ME (L)	10	85	99
	MP (R)	10	21	38
	ME (R)	2	46	65
PLI	MP (L)	0	0	0
	ME (L)	0	0	0
	MP (R)	0	0	1
	ME (R)	0	0	0

**Figure 3:**
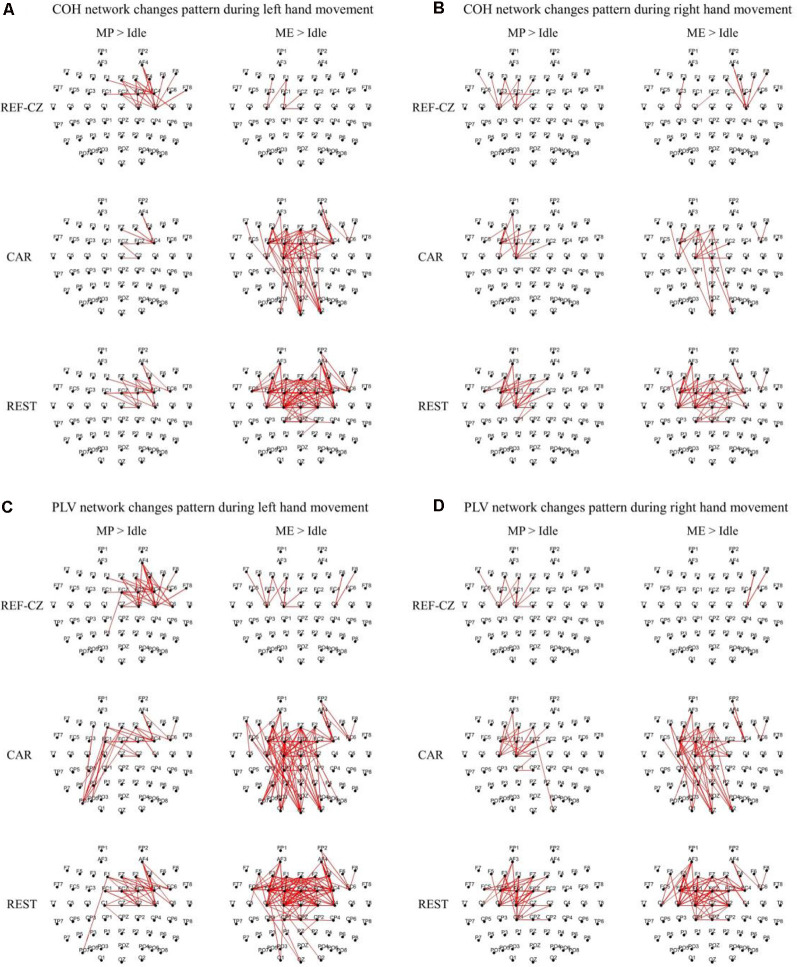
The significantly increased edges (*P* < 0.05, FDR corrected) compared to the idle stage during the movement preparation (MP) stage and movement execution (ME) stage in the beta band (13–30 Hz) from 17 subjects. The thickness of the line indicates the increase in connectivity. **(A)** Coherence (COH) network changes pattern during left hand movement. **(B)** COH network changes pattern during right hand movement. **(C)** Phase-locking value (PLV) network changes pattern during left hand movement. **(D)** PLV network changes pattern during right hand movement.

Figure 3 shows the edges of connectivity significantly increased compared to the idle stage in the PLV and COH networks with different references during left-/right-hand movements (*P* < 0.05, BHFDR). For the REF-CZ reference, the connections between the contralateral respective areas of the motor cortex can be observed during the MP stage, but there are few connections between these areas during the ME stage. For the CAR and REST references, not only can be observed the connections between the contralateral respective areas of the motor cortex during the MP stage, but also the connections between the bilateral respective areas of the motor cortex and the somatosensory cortex area during the ME stage. The connections between the respective areas of the motor cortex areas of the networks with REST reference were more than the CAR reference network. The networks with CAR reference show some non-motor cortex activities. Also, the left-hand movement had significantly more significant connectivity increases than the right-hand movement, which may be related to the fact that our subjects were all right-handed. Ipsilateral motor cortical activation could be due to additional internal effort required for left-handed movement, which is more complex than right-handed movement (Dhamala et al., 2003). This phenomenon may also be explained as the left-brain dominance for motor planning in humans (Sabate et al., 2004).

### Effect of Reference Choice on Network Measures

**Figure 4:**
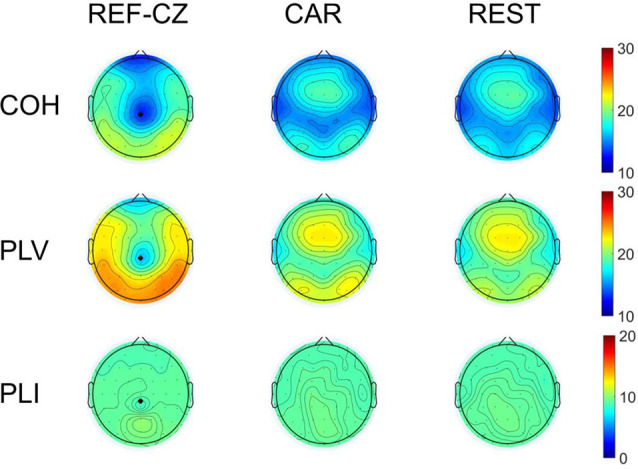
The topographic map of node degree during ME. The black crosses mark the location of the original reference REF-CZ.

To examine the differences in the local network properties of different reference networks, we calculated the degree distribution of the brain network of 17 subjects. The topographic map of the average nodal degree of the 17 subjects during the left-hand movement execution (ME) stage is shown in Figure 4. For the COH and PLV networks, the degree distribution of networks with REF-CZ reference is different from CAR reference and REST reference. Especially the degrees of the parietal near the reference electrode is very small. The degree distribution of networks with CAR and REST reference is similar, and the degree is mainly higher in the frontal. For the PLI networks, the degrees near the reference electrode of network with the REF-CZ reference is only slightly lower than the CAR and REST references. Similar results were obtained in the networks of the MP stage and ME stage of both the left and right hand.

The number of nodes with significant differences is shown in Table 2 (*P* < 0.05, *t*-test). For the COH and PLV networks, the degrees of many nodes of networks with different references are significantly different. The networks with REF-CZ reference differ greatly from networks with the CAR and REST reference, while the difference between the CAR and REST reference networks is relatively small. For the PLI network, only a few nodes have significant differences between networks with different references.

**Table 2:** The number of nodes with significant differences in node degree during the MP stage and ME stage.

Connectivity	Movement stage	The number of significantly different nodes (*P* < 0.05, *t*-test)		
		REF-CZ vs. CAR	REF-CZ vs. REST	CAR vs. REST
COH	MP (L)	43	43	25
	ME (L)	45	42	26
	MP (R)	42	43	21
	ME (R)	45	43	27
PLV	MP (L)	40	41	21
	ME (L)	43	41	22
	MP (R)	41	41	21
	ME (R)	45	44	23
PLI	MP (L)	2	1	2
	ME (L)	1	0	1
	MP (R)	1	1	1
	ME (R)	0	0	1

**Figure 5:**
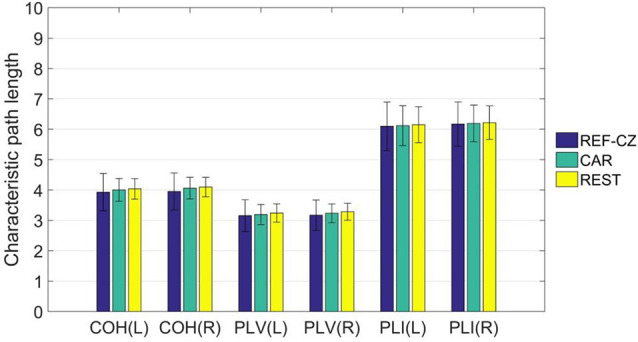
The mean (±SD) values of the characteristic path length of functional networks with different references.

For global network properties, we calculated the characteristic path length of the brain network of the 17 subjects. By comparing the characteristic path lengths among different reference networks, no significant differences were found. The average characteristic path length of the left-hand ME stage is shown in Figure 5.

## Discussion

To find a better reference electrode for hand movement task studies based on EEG, we constructed the functional networks of ten subjects with REF-CZ, CAR, and REST references. We studied the influence of the reference choice on the results of network analysis and graph theory indicators.

Many studies based on brain imaging technology had found that the interactions of cortico-motor networks, including the primary motor cortex (M1), the supplementary motor area (SMA), the premotor cortex (PMC), and parietal cortex, are strengthened during the MP stage and ME stage, and the networks showed contralateral advantage (Jiang et al., 2004; Wheaton et al., 2005; Wu et al., 2011; Popovych et al., 2016). The interaction of these areas during the MP was related to the planning of movement, and similar interaction could also be observed in motor imagery (MI; Sabate et al., 2004; Hanakawa et al., 2008). SMA and PMC were responsible for the selection, planning and some aspects of motor control for movement or the direct control of some movements and more involved in planning or preparing for movement than M1 (Weinrich and Wise, 1982; Schluter et al., 1998; Picard and Strick, 2003; Nguyen et al., 2014). The motor cortex showed stronger connectivity during the ME stage than the MP stage. M1, which was responsible for the execution of movement, played an important role in movement networks during the ME stage (Jiang et al., 2004). However, areas such as PMC and SMA also contributed to the execution of movement. One study showed that the coupling parameters among SMA, PMC and M1 increased with recovery and predicted a better outcome in stroke patients suffering from hand motor deficits (Rehme et al., 2011). Besides, the parietal and sensory cortices were also activated during hand movements (Okuda et al., 1995; Christoph et al., 2001), and showed increased cortical coupling with the PMC and SMA (Wheaton et al., 2005).

We confirmed that the choice of reference electrode significantly influences the network analysis results. For the REF-CZ reference, we could not observe obvious synchronized activity between the respective areas of the motor cortex during the ME stage where the M1 activity dominates, most network connectivity increases are overwhelmed by reference effects. The main reason for this phenomenon is that the M1 is close to REF-CZ reference, and the electrical activity could be conducted to the reference channel. The amplitude and phase information of the channel signals in M1 are lost, resulting in low measured synchronization and difficulty in distinguishing its changes. Therefore, the original REF reference is not a good choice. Both the CAR and REST references achieve much better results than the REF-CZ reference. The significant increase in connectivity between the respective areas of the motor cortex (i.e., PMC, SMA, and M1) can be observed from these two reference networks, and a clear contralateral advantage can be observed. Compared with the CAR reference, the REST reference network shows more pronounced activity in the motor cortex. The networks with CAR reference show a lot of occipital activities. This phenomenon has not been supported by relevant electrophysiological evidence during voluntary movement and our experiments did not involve any visual stimuli. It may show that the performance of the REST reference is more similar to real brain activity than CAR on scalp EEG functional network analysis.

For graph theory indicators, our results show that the local properties of the network are also influenced by different references. Beta rhythm is associated more with motor cortical function, and tend to show maximum power in the frontal and central areas (Hari and Salmelin, 1997). As mentioned above, the synchrony between the respective areas of the motor cortex will increase during the movement task. However, the degrees of networks of these areas with REF-CZ reference is small because the reference electrode is too close to the active areas. Since the degree of any node is related to all nodes in the network, the overall degree distribution will be affected by the reference effect. This reference effect is greatly reduced in the network with CAR and REST reference. As for the global property of the network, the results among different references are not significantly different. The characteristic path length, as a global network attribute, reflects the overall information transmission efficiency of the network (Bassett and Bullmore, 2006). Although different references may significantly affect the connectivity between some nodes, they are still not enough to greatly change the overall performance of the network.

For all three connectivity measures, different connectivity calculation methods are affected by reference choice. In this study, due to the lack of meaningful PLI results, the connectivity results of PLI could not be compared. The degree distribution of PLI networks is least affected by the reference. This was expected because PLI only retains non-zero lag correlation that is more likely to reflect the true correlations of underlying sources and discards zero-lag correlation that may be affected by volume conduction and reference effects (Stam et al., 2007). Thus, the PLI is theoretically much less affected by the influence of common sources and active reference electrodes. However, there are the risks of missing functionally meaningful correlations at zero lag in this approach. Compared to COH, PLV uses only phase information and no amplitude information. Therefore, PLV is slightly less affected by reference effects than COH.

In previous studies of the effect of reference electrodes on RP, the performance of the CAR reference and the REST reference has similar results (Hu et al., 2017). In this study of the influence of reference electrodes on motor functional brain networks, REST references have shown superior performance to CAR. This also illustrates that the phase information used in functional network connectivity calculations is more sensitive to the impact of reference effects than the amplitude information.

## Conclusions

On the one hand, our study provides evidence for the reference selection in the construction of the scalp EEG network during movement tasks. On the other hand, the results further confirm that the EEG reference plays an important role in data analysis in neuroscience. For the study of movement Scalp EEG functional networks, it is very important to choose the appropriate reference electrode. Our results show that the reference has a great impact on the connectivity changes of functional networks and graph theory indicators in hand movement tasks. Researchers may obtain different conclusions when functional networks are constructed with different references. In particular, online recording references located near the motor areas can lead to large biases. According to our conclusion, the REST and CAR reference can greatly reduce the effect of the online recording reference location, while REST may be slightly better than CAR.

## Data Availability Statement

The raw data supporting the conclusions of this article will be made available by the authors, without undue reservation.

## Ethics Statement

The studies involving human participants were reviewed and approved by the local ethics committee for the Protection of Human Subjects at Zhengzhou University. Participants provided their written informed consent to participate in the study.

## Author Contributions

LZ: conceptualization, methodology, software, and writing—reviewing and editing. PW: conceptualization, methodology, software, and writing—original draft preparation. RZ: conceptualization, methodology, and data curation. LS: investigation, funding acquisition, and supervision. JG: investigation, supervision, and validation. MC: investigation, and supervision. YH: conceptualization, project administration, funding acquisition, investigation, and supervision.

## Conflict of Interest

The authors declare that the research was conducted in the absence of any commercial or financial relationships that could be construed as a potential conflict of interest. The reviewer PX declared a past co-authorship with one of the authors RZ to the handling Editor.

## Footnotes

^1^http://www.neuro.uestc.edu.cn/REST/
